# IGF-1C domain–modified hydrogel enhanced the efficacy of stem cells in the treatment of AMI

**DOI:** 10.1186/s13287-020-01637-3

**Published:** 2020-03-26

**Authors:** Yong Yao, Liang Yang, Li-feng Feng, Zhi-wei Yue, Nian-huan Zhao, Zongjin Li, Zuo-xiang He

**Affiliations:** 1 Nankai University School of Medicine, Tianjin, China; 2 Department of Nuclear Medicine, The 2nd Clinical Medical College (Shenzhen People’s Hospital) of Jinan University, Shenzhen, Guangdong China; 3 Department of Pharmacology, School of Medicine, Nankai University, Tianjin, China; 4 The Key Laboratory of Bioactive Materials, Ministry of Education, the College of Life Science, Nankai University, Tianjin, China; 5 Department of Nuclear Medicine, Huangshi Central Hospital, Affiliated Hospital of Hubei Polytechnic University, Edong Healthcare Group, Huangshi, China; 6 Hubei Key Laboratory of Kidney Disease Pathogenesis and Intervention, Huangshi, China; 7 Henan Key Laboratory of Medical Tissue Regeneration, Xinxiang Medical University, Xinxiang, China; 8 Department of Nuclear Medicine, Beijing Tsinghua Changgung Hospital, School of Clinical Medicine, Tsinghua University, Beijing, China

**Keywords:** Human placenta–derived mesenchymal stem cells, CS-IGF-1C hydrogel, Acute myocardial infarction, Angiogenesis, Bioluminescence imaging

## Abstract

**Background:**

Due to the low survival rate of cell transplantation, stem cell has not been widely used in clinical treatment of acute myocardial infarction (AMI). In this study, we immobilized the C domain peptide of insulin-like growth factor-1 on chitosan (CS-IGF-1C) to obtain bioactive hydrogel. The purpose was to investigate whether CS-IGF-1C hydrogel incorporated with human placenta–derived mesenchymal stem cells (hP-MSCs) can boost the survival of hP-MSCs and enhance their therapeutic effects.

**Methods:**

hP-MSCs, which continuously expressed green fluorescent protein (GFP) and firefly luciferase (Fluc), were transplanted with CS-IGF-1C hydrogel into a mouse myocardial infarction model. Cell survival was detected by bioluminescence imaging (BLI), and cardiac function was measured by echocardiogram. Real-time PCR and histological analysis were used to explore the therapeutic mechanism of CS-IGF-1C hydrogel.

**Results:**

CS-IGF-1C hydrogel could induce the proliferation of hP-MSCs and exert anti-apoptotic effects in vitro. The Calcine-AM/PI staining results showed that hP-MSCs seeded on CS-IGF-1C hydrogel could protect neonatal mouse ventricular cardiomyocytes (NMVCs) against oxidative stress. It was observed by BLI that CS-IGF-1C hydrogel injected into ischemic myocardium could improve the survival rate of hP-MSCs. Histology analysis indicated that co-transplantation of the CS-IGF-1C hydrogel and hP-MSCs could increase angiogenesis, reduce collagen deposition, ameliorate left ventricular expanded, and further promote the recovery of cardiac function. Besides, we found that the inflammatory response was inhibited and the expression of apoptosis-related genes was downregulated by CS-IGF-1C hydrogel.

**Conclusions:**

CS-IGF-1C hydrogel provides a conducive microenvironment for cells and significantly boosts the survival of hP-MSCs in mouse myocardial infarction model, which suggest that it may be a potential candidate for prolonging the therapeutic effect of hP-MSCs during AMI.

## Introduction

Acute myocardial infarction (AMI) is one of the leading causes of heart failure, which seriously affect human life expectancy and quality of life because of its high incidence and mortality [1, 2]. The current treatment strategies include drug therapy and surgical interventional therapy. Because the regeneration capacity of adult myocardium is very limited, the effects of these treatments are not satisfactory [3, 4].

Stem cell transplantation brings a new opportunity for the treatment of myocardial infarction. Stem cell therapy is believed to be a potent strategy to treat AMI via secreting paracrine factors [5, 6]. However, low retention and survival after stem cell transplantation significantly reduce its therapeutic effect [7]. Therefore, improving cell survival is particularly important for stem cell treatment of AMI.

In order to provide scaffolds for transplanted cell anchorage and provide favorable niches for cells, some synthetic biomaterials simulating in vivo microenvironment were designed [4, 8]. Recently, many studies have demonstrated that hydrogel, as a cell carrier, injected into infarcted myocardium together with stem cells can effectively improve the survival of stem cells [9, 10]. Similar to natural tissue, chitosan hydrogel is a good sustained-release carrier, which can protect cells from being cleared by the host immune system [11, 12].

IGF-1 has a variety of effects such as promoting proliferation, anti-apoptosis, and inducing angiogenesis. It could increase the graft size and improve cell transplantation efficacy in AMI model [13]. Moreover, Haider et al. conformed that IGF-1 transgenic expression can induce a large number of stem cell mobilization through SDF-1alpha signal and promote angiogenesis in the myocardial infarcted area [14]. Thermosensitive IGF-1C hydrogel is a kind of polymer material with good biocompatibility, which can simulate extracellular matrix in vivo and provide a suitable microenvironment for stem cell migration, retention, and proliferation. With the progress of damage repair, hydrogel can be slowly degraded and replaced by repaired tissue. More importantly, the use of IGF-1C hydrogel to encapsulate stem cells has the advantages of low cost, simple operation, low requirements for instrument, and strong maneuverability, so it has a good prospect in clinical application. Feng et al. demonstrated that IGF-1C hydrogel could provide niche support and boost the survival of transplanted adipose-derived mesenchymal stem cells, ultimately improving the therapeutic effect in a mouse acute kidney injury model [12]. In this study, we try to identify whether immobilized C domain peptide of IGF-1 can enhance the effect of chitosan hydrogel for the survival rate of MSCs in myocardial infarcted area.

Another key issue after MSC transplantation is how to track cell migration and survival in vivo. Molecular imaging can dynamically and quantitatively track biological processes, which provides the possibility for real-time monitoring of treatment response and non-invasive evaluation of treatment mechanisms [15, 16]. Compared with other imaging methods, the bioluminescence imaging (BLI) system can real-time and quantitatively monitor the migration, differentiation, and proliferation of transplanted cells. In addition, BLI system has high sensitivity, which makes BLI technology more widely used in stem cell research [17, 18].

In this study, we speculated that thermosensitive bioactive CS-IGF-1C hydrogel could provide a conducive microenvironment for hP-MSCs to boost cell survival and enhance their therapeutic capacity. To test this hypothesis, hP-MSCs were transplanted with CS-IGF-1C hydrogel into a mouse myocardial infarction model and the therapeutic effects were evaluated. To accurately track the fate of hP-MSCs, dual reporter genes green fluorescence protein (GFP) and firefly luciferase (Fluc) were introduced in this study for monitoring cell survival in vivo in mice.

## Materials and methods

### CS-IGF-1C hydrogel preparation

IGF-1C immobilized thermosensitive CS hydrogel was prepared according to the previously reported method [19, 20]. In short, the CS-IGF-1C powder was dissolved in 0.1 M acetic acid, filtered and sterilized, and then placed in an ice bath to prepare a stock solution of 2% CS-IGF-1C. The 50% β-glycerophosphate solution (Sigma-Aldrich, Milwaukee, WI, USA) was prepared with tri-distilled water and then sterilized with a 0.2-μm filter. The β-glycerophosphate solution was added into the CS-IGF-1C solution in a volume ratio of 5:1 under continuous stirring in an ice bath for 0.5 h. After incubation at 37 °C for 5–10 min, the mixed solution could turn into hydrogel. The CS hydrogel was prepared with the same protocol.

### Cell culture

hP-MSCs were isolated according to the previously reported method [21]. In short, the pregnant women had the informed consent before donating the placentas. hP-MSCs from chorionic villi were collected. The entire tissue was cut into 1–2-mm pieces and then digested in 0.2 mg/ml collagenase IV for 90 min. Processed tissue was squeezed through a 100-μm cell filter to remove cell aggregates. hP-MSCs were cultured in DMEM/F12 medium (Gibco, Grand Island, NY) with 1% 100 U/ml penicillin-streptomycin, 1% non-essential amino acid, and 10% fetal bovine serum (FBS, HyClone, Australia). The number of passage of MSC used in this study was the fifth generation. To monitor cells in vivo, hP-MSCs were transduced with a self-inactivating lentiviral vector driving firefly luciferase (Fluc) and GFP reporter gene. In addition, to see whether there is a positive relationship between the number of cells and the fluorescence intensity, Fluc-labeled HP-PMSC cells were seeded into six-well culture plate according to the cell concentration gradient (0, 0.125 × 10^5^, 0.25 × 10^5^, 0.5 × 10^5^, 1 × 10^5^, 2 × 10^5^) and cultured in an incubator. After 24 h, it was determined by BLI. Human umbilical vein endothelial cells (HUVECs) were bought from ATCC (ATCC, Manassas, VA) and cultured as before.

### Cell proliferation analysis

To evaluate the beneficial effects of CS-IGF-1C hydrogel on cell proliferation, CS-IGF-1C or CS hydrogel was added to each well of a 12-well cell culture plate. Then, hP-MSCs were seeded onto cell culture plates at a density of 1 × 10^5^ cells/well with 300 U/l β-glycerophosphate solution in the medium. At different time points (24 h, 48 h, and 72 h), Imaging System IVIS Lumina (Xenogen Corporation, Hopkinto, MA) was employed to measure the number of survival cells. The proliferation of cells was judged by fluorescence intensity. And the fluorescence intensity was expressed in units of photons/second/cm^2^/steradian (p/s/cm^2^/sr).

As for the experiment of Ki-67 staining, 2 × 10^4^ hP-MSCs were seeded on the cover slips in 24-well cell culture plate. The cells were incubated with CS-IGF-1C or CS hydrogel and cultured for 24 h. The control group was PBS. Then, the cells on cover slips were stained with immunofluorescence (Rabbit anti-Ki-67, Abcam). The photographs were taken under a fluorescence microscope (Nikon, Tokyo, Japan). Image J software was used to measure the percent of Ki-67^+^ cells.

### Cell apoptosis assay

In order to investigate whether CS-IGF-1C hydrogel can rescue H_2_O_2_-induced injury, hP-MSCs (5 × 10^5^ cells/well) were seeded onto 6-well cell culture plates and cultured for 12 h. Then, culture medium was changed to complete medium which contained 500 μM hydrogen peroxide (H_2_O_2_), to simulate the oxidative damage caused by myocardial infarction in vivo. The treatment group was added CS-IGF-1C or CS hydrogel separately. PBS was used as control. Four hours later, cell survival was determined by BLI. To explore the mechanisms involved in this apoptosis action, real-time PCR and Western blot were used to measure the expression of apoptosis-related genes and protein in hP-MSCs.

### Scratch wound healing assay

In order to evaluate the ability to promote migration, HUVECs were seeded into 24-well cell culture plate. When cell confluence reached over 85% or more, a scratch wound was made in cell monolayers with a 10-μl micropipette tip. HUVECs can grow for additional 12 h with hP-MSCs by using a semipermeable membrane of Transwell insert (pore size, 8.0 μm) (Corning Company), which separates the two types of cells but allows secreted factors to spread to each other. PBS was used as control. At 0 and 12 h, pictures of five areas were taken with an inverted microscope (Olympus, Lake Success, NY). The migration distance was measured using the Image J software.

### Neonatal mouse ventricular cardiomyocyte isolation and culture

Primary neonatal mouse ventricular cardiomyocytes (NMVCs) were prepared from the hearts of 24–48-h-old C57BL/6 mice. The protocol for isolating and culturing NMVCs was as described previously [22]. In short, the hearts of the neonatal mouse were rapidly removed and left ventricles were cut into 1–2 mm^3^ small cubes. Then, heart tissues were dissociated in 0.25% trypsin at 37 °C. After myocardial tissues were digested to disappear, tissue fluid was centrifuged at 2500*g* for 5 min to collect the cell suspension. The cells in suspension were then resuspended in Dulbecco’s modified Eagle’s medium (DMEM; HyClone, Logan, UT) supplemented with penicillin (100 U/ml)/streptomycin (100 U/ml) and 10% fetal bovine serum (Gibco, Grand Island, NY), and cultured at 37 °C in 5% CO_2_ incubator. After 90 min, fibroblast adhesion was performed and the cell suspension was plated into a 6-well plate at 3 × 10^5^ cells per well.

### Co-culturing of hP-MSCs and NMVCs

To observe the protective effects of CS-IGF-1C hydrogel, we established a co-culture model of hP-MSCs and NMVCs in vitro as previously described [23, 24]. In short, hP-MSCs and NMVCs were indirectly co-cultured in a 1:10 ratio by using a semipermeable membrane of Transwell insert (pore size, 8.0 μm) (Corning Company), which separates two types of cells but allows the diffusion of secreted factors. hP-MSCs (1 × 10^4^cells/well) were seeded onto Transwell insert. On the one hand, co-culture was incubated with DMEM supplemented with 10% fetal bovine serum at 37 °C and 5% CO_2_ for 12 h. Then, culture medium was changed to complete medium which contained 500 μM H_2_O_2_, to simulate the oxidative damage caused by myocardial infarction in vivo. On the other hand, NMVC ischemia model was performed as previously described [25]. Briefly, NRVCs were cultured in a serum-free DMEM in an anaerobic chamber with 95% N_2_/5% CO_2_ for 8 h and reperfusion at 37 °C for 1 h. Then, co-culture was maintained with DMEM supplemented with 10% fetal bovine serum at 37 °C and 5% CO_2_ for 12 h. Next, Calcein-AM/PI double staining was performed. NRVCs were incubated in PBS containing 1 mg/ml propidium iodide (PI) and 2 mg/ml Calcein-AM at 37 °C for 10 min. Then, pictures were taken with a fluorescence microscopy.

In order to evaluate the effect of CS-IGF-1C hydrogel on cell viability, Cell Counting Kit-8 (CCK-8) (MedChem Express, Monmouth Junction, NJ) was used following the manufacturer’s protocol. In short, after 12 h of co-culture, hydrogen peroxide was added to the culture medium. After incubation for 4 h, each well was replaced with normal culture medium which contained 10% CCK-8 solution and incubated at 37 °C for 3 h. Cell viability was determined at 450 nm.

### Transplantation of hP-MSCs with hydrogel

The C57BL/6 background transgenic mice (8–10 weeks old) expressed Fluc under the promoter of vascular endothelial growth factor receptor 2 (VEGFR2-luc), from Sibeifu Corporation (Beijing, China). Mice were raised under a specific pathogen free (SPF) animal area at the Animal Facility of Nankai University. The operation and the experimental protocol of the research animals were performed in accordance with the guidelines of Use Committee and Nankai University Animal Care, which were in accordance with the guidelines for animal care approved by the National Institutes of Health (8th edition, 2011).

For in vivo transplantation, CS-IGF-1C or CS hydrogel was prepared according to the protocol described above. As mentioned earlier, the left anterior descending branch of the coronary artery was ligated to make a model of myocardial infarction [26]. After ligation, 5 × 10^5^ hP-MSCs were suspended in 20 μl CS-IGF-1C hydrogel, CS hydrogel, or PBS. Then, cell suspension was injected into two positions adjacent to the infarcted areas. The same procedure was performed without ligation for sham-operated control (fifteen mice in each group).

### Bioluminescence imaging analysis

In the ex vivo experiments, Fluc-labeled hP-MSCs in culture plates were detected by Imaging System IVIS Luminar System (Xenogen Corporation, Hopkinto, MA). The average radiance of the region of interest (ROI) was measured by BLI. As for in vivo experiments, the in vivo Imaging System IVIS Luminar was used to detect the expression level of Fluc every other day. Mice were anesthetized, and then, 20 μl d-Luciferin was injected through the fundus. Then, they were imaged immediately for 1–5 min using IVIS Lumina Imaging System.

### Assessment of cardiac function

To assess cardiac function, echocardiographic examination was performed on day 30. Mice were anesthetized. The Vevo® 2100 System equipped with a 30-MHz transducer (FUJIFILM VisualSonics, Inc. Toronto, Canada) was used to detect cardiac function. Left ventricular internal diameter at end-systole (LVIDs), left ventricular internal diameter at end-diastole (LVIDd), left ventricular fraction shortening (LVFS), and left ventricular ejection fraction (LVEF) were analyzed using the Vevo 2100 workstation software.

### Histochemical and immunofluorescence staining

On days 7, 14, and 30, mice were executed to harvest heart samples. Then, the heart tissues were fixed with 4% formaldehyde and embedded in paraffin. Then, paraffin tissues were cut into 6–8-μm-thick sections, which were stained with Masson’s staining and hematoxylin and eosin (HE). The ratio of collagen area to the total left ventricular area was taken as the collagen content. Image J software was used to measure the area of collagen and LV.

For immunofluorescent staining, the heart tissues were embedded into OCT compound (Sakura Finetek, Tokyo, Japan). Then, the fixed tissue was cut into 6–8-μm-thick sections. The sections were incubated with primary antibody against CD31 (rat anti-mouse; Abcam, Cambridge, MA) over night, and then, AlexaFluor 594 goat anti-rat IgG (Invitrogen, Grand Island, NY) was dripped. Cell nuclei were counterstained with 6-diamidino-2-phenylindole (Southern Biotech, Birmingham, AL). The total amount of capillaries in the infarct area were determined in three random fields.

### Quantitative RT-PCR analysis

Total RNA from heart samples or cell were extracted with TRIzol reagent (Invitrogen, Grand Island, NY) according to the manufacturer’s protocol. Then, the total RNA was reverse transcribed into cDNA using a First-Strand cDNA Synthesis System (TransGen Biotech, China). Real-time PCR (Bio-Rad, Hercules, CA, USA) was used to quantify related gene expression levels. Relative gene expression folding changes were identified with 2^-ΔΔCt^ method, and the primer sequences used in our study are shown in Supplemental Table 1 and Table 2.

### Western blot

Cells were lysed and incubated in RIPA buffer for 30 min. BCA Protein Assay Kit (Thermo, USA) was used to measure protein concentration. The proteins were electro-blotted to a polyvinylidene fluoride (PVDF) membrane (Millipore, USA). The membrane was incubated with a primary antibody against β-Tubulin (^#^sc-166729, Santa Cruz Biotechnology, USA) and Cleaved Caspase-3 (^#^9664 s, Cell Signaling Technology, USA). Image J software was used to quantify Western blot bands. The results were expressed as fold changes normalized to β-Tubulin as an internal control.

### Statistical analysis

GraphPad Prism 5.0 software (GraphPad Prism Software Inc., San Diego, CA, USA) was used for statistical analysis of one-way ANOVA. All data were expressed as standard error of the mean (SEM). *P* values less than 0.05 were accepted as statistically significant.

## Results

### hP-MSC labeling and characterization of CS-IGF-1C hydrogel

**Fig. 1:**
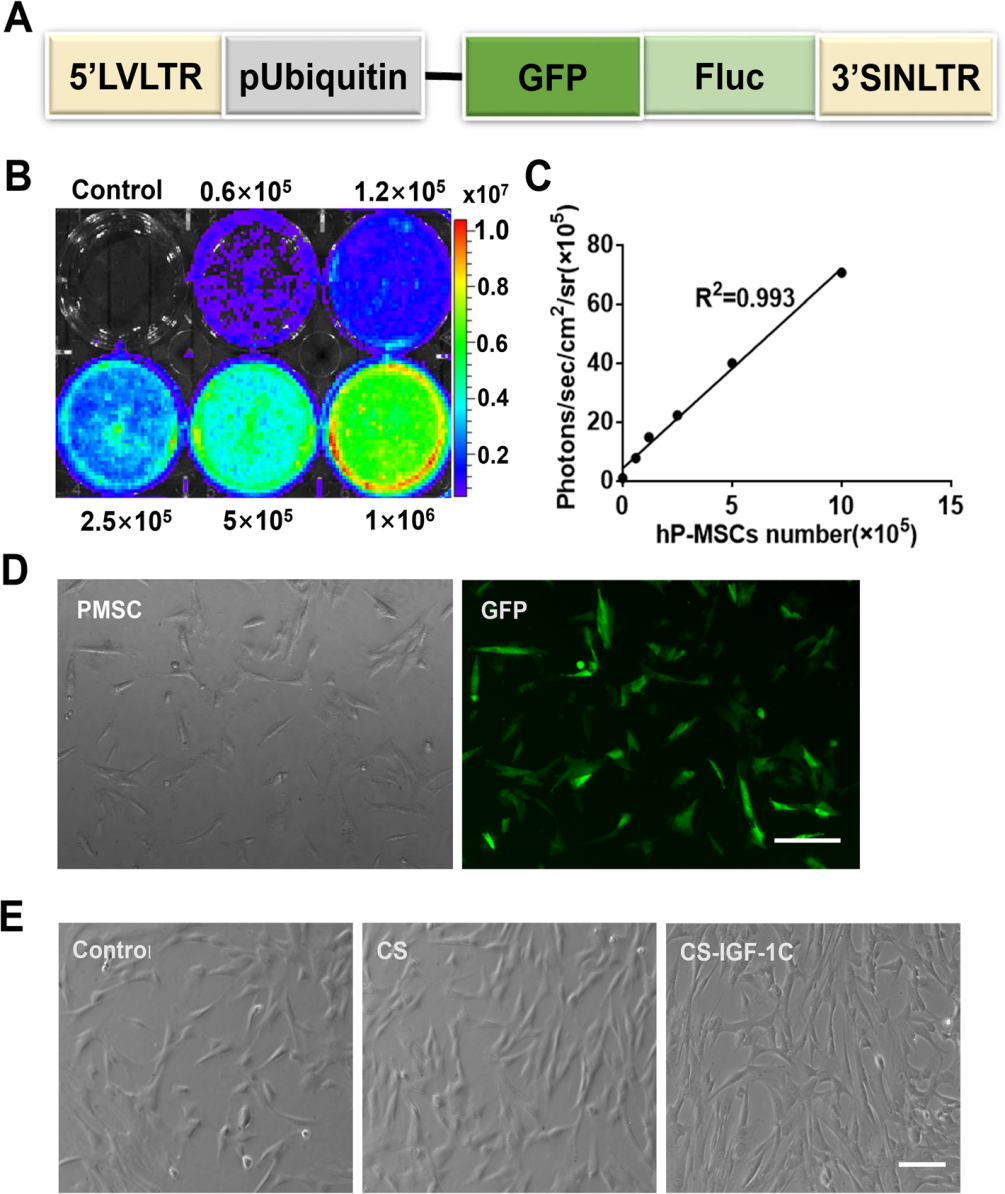
Characterization of the double fusion (DF) firefly luciferase (Fluc) and green fluorescent protein (GFP) hP-MSCs. **a** Schematic representation of the DF reporter gene containing Fluc and GFP driven by an ubiquitin promotion. **b**, **c** BLI quantification showed increasing bioluminescence signals with cell numbers (*R*^2^ = 0.993). **d** hP-MSCs are strongly positive for GFP on fluorescence microscopy. **e** CS-IGF-1C hydrogel is biocompatible in vitro. Scale bar, 100 μm

In this study, hP-MSCs continuously expressed GFP and Fluc (Fig. 1a). There is a significant linear correlation between the optical intensity of Fluc average radiance and Fluc-labeled hP-MSC number (*R*^2^ = 0.993, Fig. 1b, c). By immunofluorescence analysis, GFP was strongly expressed on hP-MSCs (Fig. 1d). CS-IGF-1C hydrogel was obtained by grafting the C domain of IGF-1 (IGF-1C) onto the side chain of CS (Supplemental Fig. 1A). The thermosensitive CS-IGF-1C hydrogel neutralized with β-glycerophosphate was liquidized at 4 °C and changed into hydrogel at 37 °C (Supplemental Fig. 1B). It showed better biocompatibility and bioactivity of CS-IGF-1C hydrogel, which has a good effect on cell proliferation (Fig. 1e).

### Proliferative and protective effects of CS-IGF-1C hydrogel in vitro

**Fig. 2:**
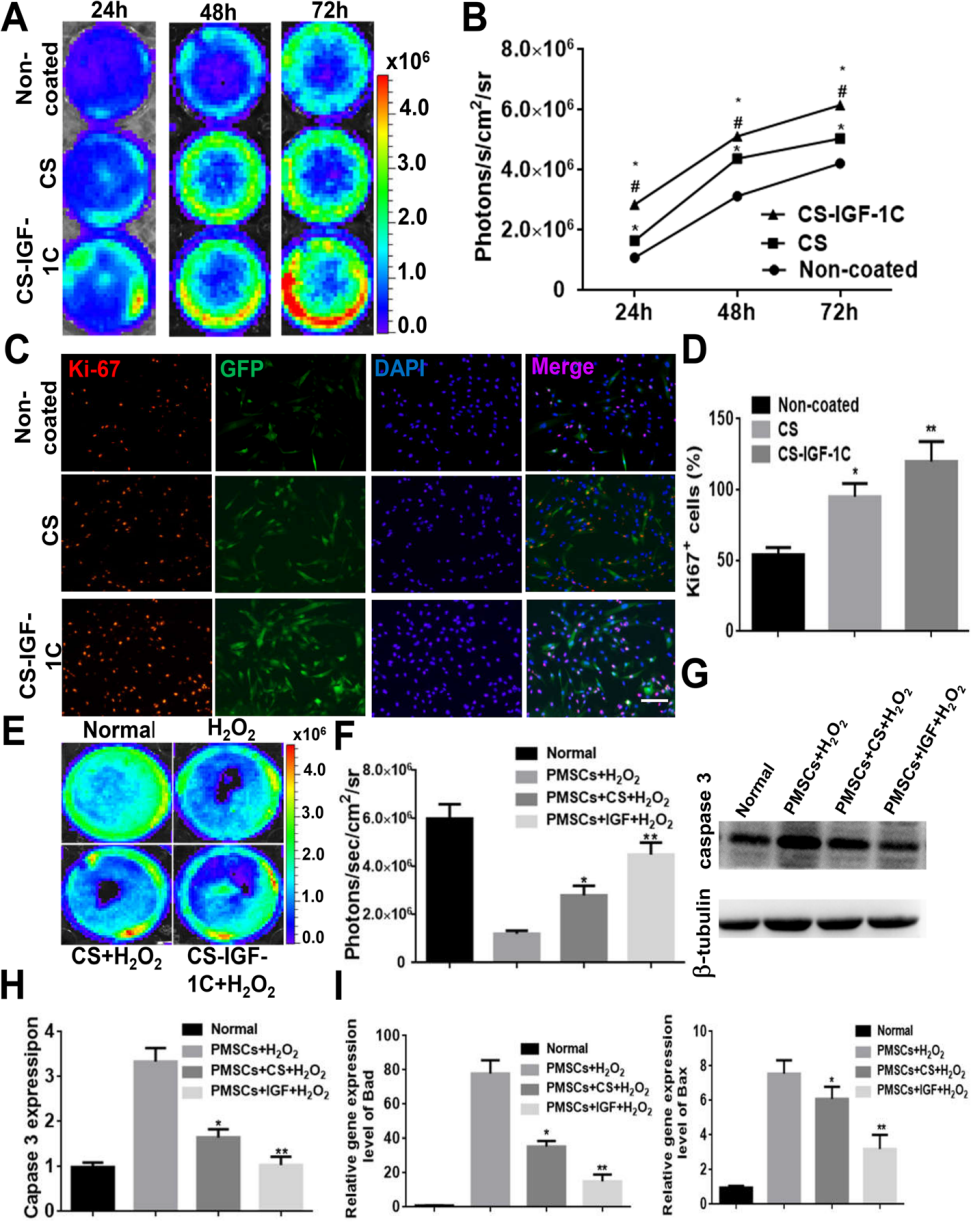
Proliferative and protective effects of CS-IGF-1C hydrogel in vitro. **a** BLI exhibited that CS-IGF-1C hydrogel enhanced the proliferation of hP-MSCs. **b** Quantitative analysis of BLI signals. The signal activity expressed as photons/second/cm^2^/steradian. *P < 0.05 versus non-coated; ^#^*P* < 0.05 versus CS. **c** Representative images showed the proliferation (Ki-67, red) of hP-MSCs (GFP, green) incubated with CS or CS-IGF-1C hydrogel for 24 h. The bar represented 100 μm. **d** Quantification of the proliferation index of hP-MSCs performed by CS or CS-IGF-1C hydrogel. *P < 0.05 versus non-coated; **P < 0.01 versus non-coated. **e** Anti-apoptotic effects of CS-IGF-1C hydrogel. BLI revealed that CS-IGF-1C hydrogel protected hP-MSCs under oxidative condition. **f** Quantification of BLI signals displayed a significant amelioration when cultured on CS-IGF-1C hydrogel-coated plates after H_2_O_2_ treatment. *P < 0.05 versus non-coated; **P < 0.01 versus non-coated. **g** Caspase-3 and Tubulin expressions were detected by Western blots. **h** Quantification of Caspase-3 expression. **i** Real-time quantitative PCR analysis of apoptosis-related gene expression of hP-MSCs after treated with H_2_O_2_ for 4 h. All data were shown as means ± SEM in triplicate assays. *P < 0.05 versus PMSCs + H_2_O_2_; **P < 0.01 versus PMSCs + H_2_O_2_

After CS-IGF-1C hydrogel or CS treatment, BLI was used to measure cell proliferation at 24 h, 48 h, and 72 h, respectively (Fig. 2a). hP-MSCs cultured on CS-IGF-1C hydrogel-coated plates expanded faster than cultured on non-coated or CS-coated plates (*P* < 0.05, Fig. 2b). By using proliferating cell nuclear antigen (Ki-67) staining, we investigated the effect of CS-IGF-1C hydrogel on promoting cell proliferation (Fig. 2c). From the results, the percentage of Ki-67^+^ cells increased significantly after using CS-IGF-1C hydrogel for 24 h (*P* < 0.01, Fig. 2d).

On the other hand, oxidative stress can induce cell apoptosis, which is the main reason for low survival rate of stem cells after transplantation into ischemic tissue [16]. Therefore, we studied whether CS-IGF-1C hydrogel has an antioxidant effect under oxidative condition. H_2_O_2_ was used to treat hP-MSCs, and BLI was used to determine cell survival. The results revealed that the signal was significantly decreased after treatment with H_2_O_2_. Compared with CS hydrogel-coated and non-coated plates, the cell survival rate was significantly improved when cultured on CS-IGF-1C hydrogel-coated plates (*P* < 0.01, Fig. 2e, f).

To determine the mechanism of this protective effect, we examined the expression of apoptosis-related genes in hP-MSCs after H_2_O_2_ treatment. The apoptosis protein Caspase-3 expression was obviously increased in hP-MSCs after H_2_O_2_ treatment. The expression of apoptotic protein Caspase-3 was significantly decreased in CS hydrogel coating, which was further reduced after treatment with CS-IGF-1C hydrogel (*P* < 0.01, Fig. 2g, h). The same results were obtained for Bad and Bax expression (*P* < 0.01, Fig. 2i). These results indicate that CS-IGF-1C hydrogel could reduce the effect of oxidative stress on hP-MSCs.

**Fig. 3:**
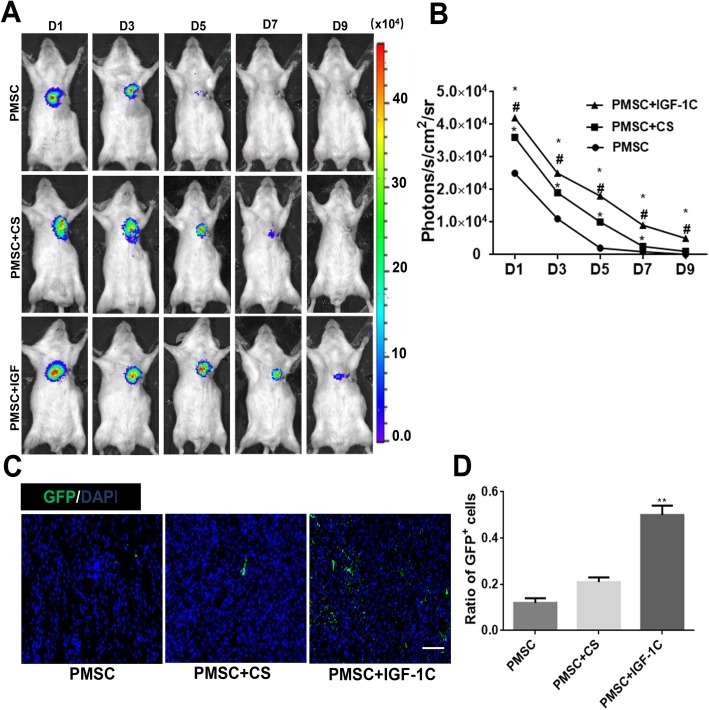
Anti-apoptotic and anti-anoxia effect of CS-IGF-1C hydrogel and hP-MSC co-transplantation on NMVCs. **a** Calcein-AM/PI staining was used to assess the influence of CS-IGF-1C hydrogel and hP-MSC co-transplantation on anti-apoptotic effect in NMVCs. **b** Quantification analysis of Calcein-AM/PI staining. *P < 0.05 versus PBS + H_2_O_2_; **P < 0.01 versus PBS + H_2_O_2_; ^#^*P* < 0.05 versus PBS + CS + H_2_O_2_. **c** Anti-anoxia effect was evaluated by Calcein-AM/PI staining. **d** Quantification analysis results of Calcein-AM/PI staining revealed that the percentage of myocardial cell death were markedly lower in the presence of CS-IGF-1C hydrogel. **P < 0.01 versus Hypoxia; ^#^*P* < 0.05 versus Hypoxia/PMSCs/CS. **e** CCK-8 results shows that NMVC survival was ameliorated in response to H_2_O_2_ after CS-IGF-1C hydrogel and hP-MSC co-transplantation. *P < 0.05 versus Normal. **f** Real-time quantitative PCR analysis of Bad gene expression of NMVCs after treated with H_2_O_2_. All data were shown as means ± SEM in triplicate assays. *P < 0.05 versus PBS; **P < 0.01 versus PBS; ^#^*P* < 0.05 versus PMSCs

Next, we assessed the influence of combined transplantation of CS-IGF-1C hydrogel and hP-MSCs on anti-apoptotic and anti-anoxia effect in NMVCs by using Calcine-AM/PI staining (Fig. 3a, c). From the results, the percentage of myocardial cell death were markedly lower in the CS-IGF-1C hydrogel group (*P* < 0.01, Fig. 3b, d). In addition, CCK-8 was used to monitor cell survival after NMVCs were treated with H_2_O_2_. As shown in Fig. 3e, NMVC survival was ameliorated in response to H_2_O_2_ after co-transplantation of hP-MSCs and CS-IGF-1C hydrogel (*P* < 0.05). To determine the protection mechanism, we measured the expression of apoptosis-related genes. The expression of Bad decreased significantly after coating with CS-IGF-1C hydrogel (*P* < 0.01, Fig. 3f). According to the above results, we considered that CS-IGF-1C hydrogel could reduce the effect of oxidative stress on NMVCs.

### Improvement of cell survival in vivo by CS-IGF-1C hydrogel

**Fig. 4:**
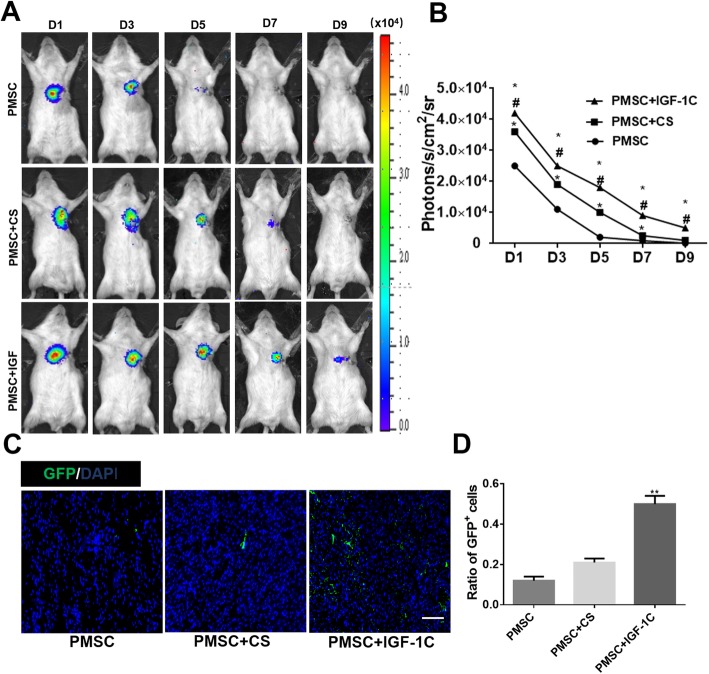
CS-IGF-1C hydrogel increased hP-MSC survival in vivo. **a** Representative BLI of mice transplanted with hP-MSCs, hP-MSCs + CS hydrogel, or hP-MSCs + CS-IGF-1C hydrogel. **b** Quantitative analysis of BLI signals showed that cell survival was improved by CS-IGF-1C hydrogel. Data are expressed as mean ± SEM. *P < 0.05 versus hP-MSCs; ^#^*P* < 0.05 versus hP-MSCs + CS. **c** Representative photomicrographs displayed the engraftment of GFP^+^ hP-MSCs delivered with CS-IGF-1C hydrogel, CS hydrogel, or PBS on day 7. Scale bar, 100 μm. **d** Quantification analysis of GFP^+^ hP-MSCs delivered with CS-IGF-1C hydrogel, CS hydrogel, or PBS. **P < 0.01 versus PMSCs

In order to evaluate whether CS-IGF-1C hydrogel can improve the survival rate of transplanted cells, hP-MSCs were tracked longitudinally with BLI analysis in mouse AMI model. On day 1 after cell transplantation, similar robust signals from the cardiac region in all groups were observed, which indicates the same number of cells was successfully delivered. Overtime, all groups experienced significant donor cell death. CS-IGF-1C hydrogel could increase cell survival rate, which could augment the therapeutic potential of hP-MSCs (Fig. 4a, b). Immunohistology also confirmed that CS-IGF-1C hydrogel increased cell engraftment. From the photomicrograph results, the effect of CS-IGF-1C hydrogel on promoting survival of GFP^+^ hP-MSCs was significantly higher than that of CS hydrogel or PBS (Fig. 4c). Moreover, the content of GFP^+^ hP-MSCs in the CS-IGF-1C hydrogel group was apparently higher than that of hP-MSCs alone or CS hydrogel (*P* < 0.01, Fig. 4d).

### Improvement of the proangiogenic capacity of hP-MSCs by CS-IGF-1C hydrogel

**Fig. 5:**
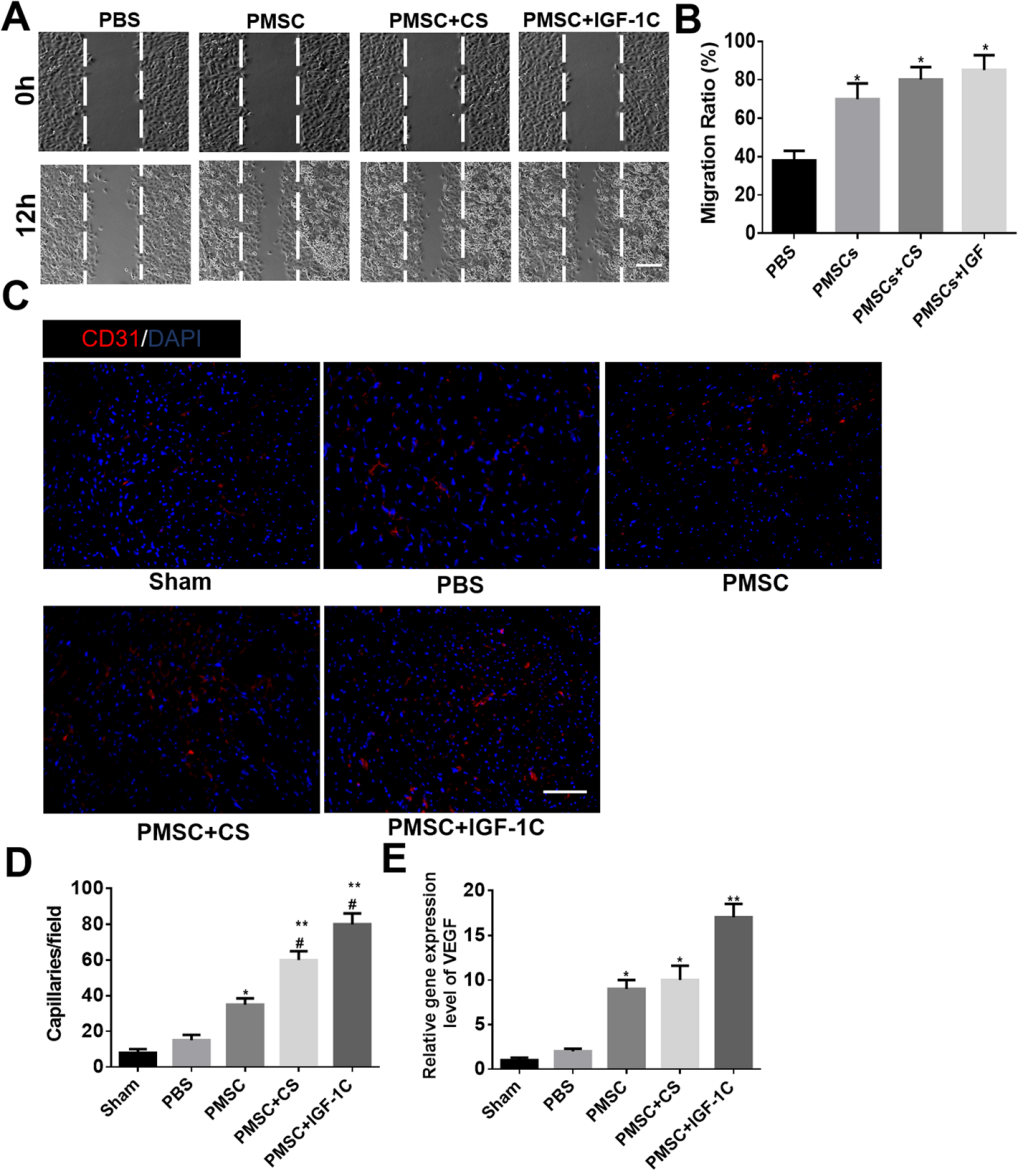
CS-IGF-1C hydrogel augmented the proangiogenic capacity of hP-MSCs. **a** Scratch wound healing assay of HUVECs treated with CS-IGF-1C hydrogel for 0 and 12 h. *P < 0.05 versus PBS; scale bar represented 200 μm. **b** The migration ratio statistics of HUVECs in each group. **c** Representative images of myocardium sections stained for CD31 (red) at day 14. Scale bar, 100 μm. **d** Quantitative analysis of CD31 immunostaining in each group. The number of capillary vessels was counted in three randomly selected areas. Data are expressed as mean ± SEM. *P < 0.05 versus PBS; **P < 0.01 versus PBS; ^#^*P* < 0.05 versus PMSC. **e** Expression levels of VEGF by RT-PCR in each group on day 14. Data are expressed as mean ± SEM. *P < 0.05 versus PBS; **P < 0.01 versus PBS

To investigate the proangiogenic potential of hP-MSCs co-transplanted with CS-IGF-1C hydrogel, scratch wound healing assay was carried out by HUVECs. After 12-h incubation of CS-IGF-1C hydrogel, the wound closure rate (85%) was higher than that of the PBS group (38%), hP-MSCs alone (70%), or CS hydrogel (80%) (Fig. 5a, b).

The proangiogenic effect of hP-MSC and CS-IGF-1C hydrogel co-transplantation was also detected in vivo in mouse AMI model. The neovascularization of injury tissues on day 14 after CS-IGF-1C hydrogel and hP-MSC co-transplantation was investigated by histologic examination. High levels of CD31, which indicate microvascular density, were expressed by the administration of CS-IGF-1C hydrogel and hP-MSCs (Fig. 5c, d). And the expression of angiogenesis gene VEGF was markedly increased by hP-MSC and CS-IGF-1C hydrogel co-transplantation (Fig. 5e). All these data suggest that CS-IGF-1C hydrogel could enhance the angiogenic effect of hP-MSCs.

### Improvement of heart function by hP-MSCs co-transplanted with CS-IGF-1C hydrogel

**Fig. 6:**
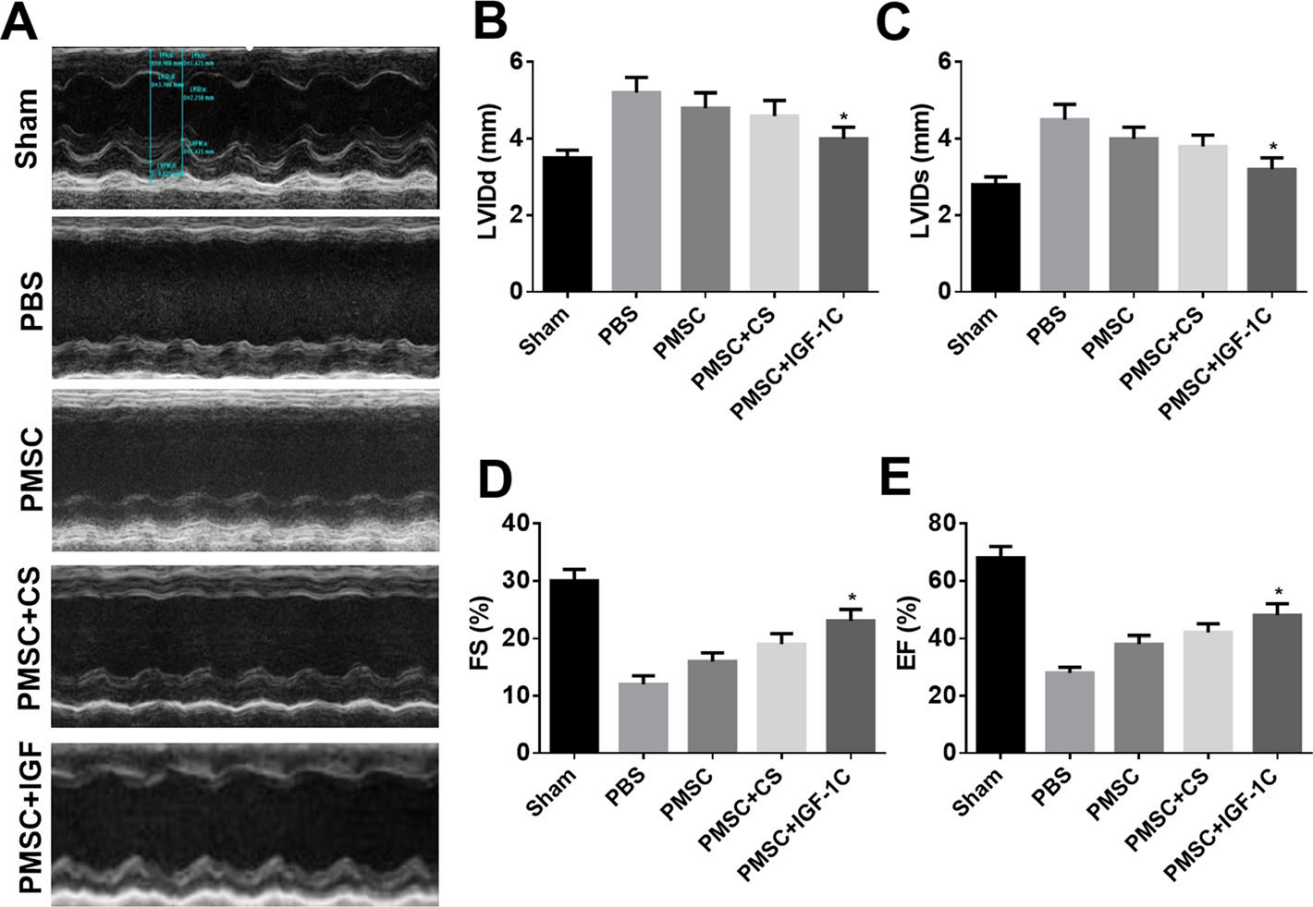
CS-IGF-1C hydrogel co-transplanted with hP-MSCs improved heart function. **a** Representative echocardiographic photos of each group at day 30. **b** LVIDd, left ventricular internal diameter at end-diastole. **c** LVIDs, left ventricular internal diameter at end-systole. **d** FS, fractional shortening. **e** EF, ejection fraction. These data suggested that CS-IGF-1C hydrogel co-transplanted with hP-MSCs could ameliorate heart function compared with other groups. *P < 0.05 versus PBS

Thirty days after cell transplantation, echocardiography was used to evaluate the left ventricular function. Compared to the sham group, the left ventricular of the other groups of mice was significantly enlarged, and the cardiac function was decreased (Fig. 6a). LVIDd and LVIDs were obviously reduced by the administration of hP-MSCs in the CS-IGF-1C hydrogel group (*P* < 0.05, Fig. 6b, c). Meanwhile, CS-IGF-1C hydrogel combined with hP-MSCs can significantly improve the LV contractile function defined by FS and EF (*P* < 0.05, Fig. 6d, e).

**Fig. 7:**
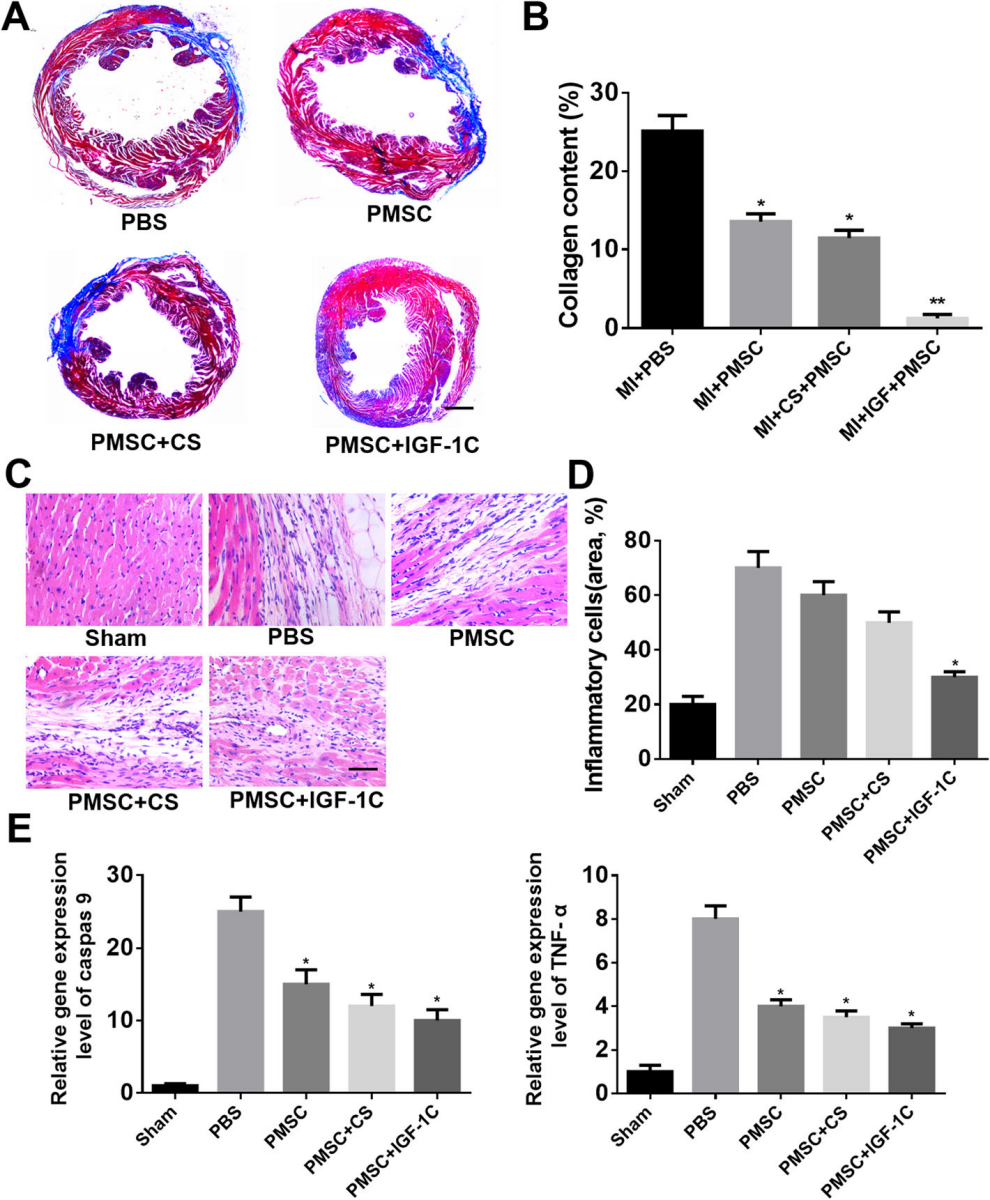
CS-IGF-1C hydrogel co-transplanted with hP-MSCs decreased collagen deposition and inflammatory cells. **a** Representative Masson’s trichrome staining of heart sections at day 30. Scale bar represents 1 mm. **b** Quantitative analysis of the area of fibrosis. *P < 0.05 versus PBS; **P < 0.01 versus PBS. **c** HE staining of heart sections in peri-infarction area on day 14. Scale bar, 100 μm. **d** Quantification of inflammatory cells. *P < 0.05 versus PBS. **e** Expression levels of caspase 9 and TNF-α by RT-PCR in each group on days 30 and 14, respectively. Data are expressed as mean ± SEM. *P < 0.05 versus PBS

Next, on the 30th day, the degree of fibrosis of myocardial tissue was detected by Masson’s staining. Significant fibrosis and left ventricular enlargement were observed in all myocardial infarction mice (Fig. 7a). The content of collagen significantly decreased in the CS-IGF-1C hydrogel group compared with the PBS group (*P* < 0.01, Fig. 7b). In addition, the injured heart tissues were examined by HE staining at day 14. The results showed that co-transplantation of CS-IGF-1C hydrogel and hP-MSCs remarkably reduced inflammatory cells in injured tissues (Fig. 7c, d). All these data indicate that co-transplantation of CS-IGF-1C hydrogel and hP-MSCs could improve the heart function and reduce left ventricular remodeling, thus saving the damaged heart.

### Molecular mechanism of saving damaged myocardium by hP-MSCs co-transplanted with CS-IGF-1C hydrogel

In order to investigate the protective mechanisms of CS-IGF-1C hydrogel, fibrosis-related genes caspase 9 and inflammatory-related factor TNF-α were detected by RT-PCR. From the results, caspase 9 and TNF-α were downregulated in murine ischemic myocardium injected with CS-IGF-1C hydrogel and hP-MSCs (*P* < 0.05, Fig. 7e). This result indicates that CS-IGF-1C hydrogel could enhance the protective effect of hP-MSCs by regulating inflammatory-related factor TNF-α and fibrosis-related genes caspase 9.

## Discussion

**Fig. 8:**
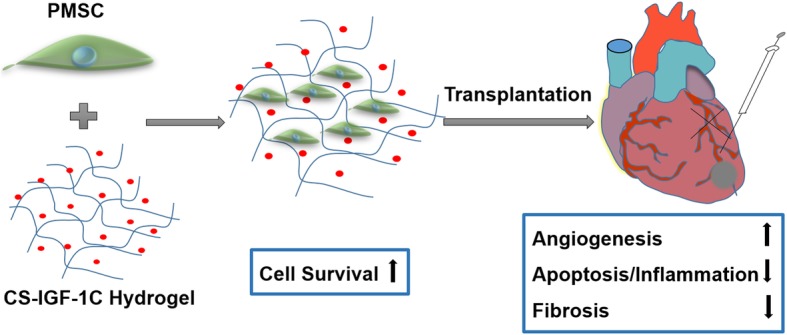
Schematic illustration of CS-IGF-1C hydrogel with hP-MSCs for treatments of acute myocardial infarction. In this study, BLI was applied for tracking the survival of hP-MSCs by Fluc imaging. CS-IGF-1C hydrogel provides a conducive microenvironment for cells and significantly improves the survival of hP-MSCs in mouse MI model, which could prolong the therapeutic effect of hP-MSCs by promoting angiogenesis, anti-apoptosis, and inhibiting fibrosis, and further promote function recovery of the damaged myocardium

In this study, our results showed that an injectable CS-IGF-1C hydrogel could provide a conducive microenvironment for hP-MSCs transplanted into the ischemic region of a mouse AMI model. We demonstrated that CS-IGF-1C hydrogel could exert proliferative, anti-apoptotic, and proangiogenic effects on cultured hP-MSCs. When transplanted into the ischemic myocardium in mice, CS-IGF-1C hydrogel could increase survival of hP-MSCs, increase angiogenesis, reduce collagen deposition, ameliorate left ventricular expanded, and further promote the recovery of cardiac function (Fig. 8). Moreover, the enhanced hP-MSC survival by CS-IGF-1C hydrogel could be directly visualized by the advanced in vivo BLI imaging technique.

Cardiac function after myocardial infarction could be improved by stem cell transplantation, but stem cell therapy has not been widely used in clinical practice due to the low survival rate of transplanted cells. The main causes of donor cell death after transplantation may be lack of matrix support, oxidative stress, and inflammation. In this study, CS hydrogel combined with immobilized IGF-1C peptide could provide a conducive niche for cell-matrix anchorage and significantly boost the engraftment and survival of transplanted hP-MSCs in vivo, which may be a potential candidate for prolonging the therapeutic effect of hP-MSCs during AMI. Overall, we provide an effective way to maintain hP-MSC-induced tissue protection instead of multiple administrations, which may be easier to operate and more secure.

The extent of infarction in acute myocardial infarction is largely determined by the inflammatory response. Adverse remodeling of the left ventricle after myocardial infarction is associated with a persistent pro-inflammatory response [27]. Strategies modulating the inflammatory cascade may not only hold promise as protective measures to prevent remodeling, but may also prove crucial in realizing the goal of preventing of post-infarction heart failure [28, 29]. Luger et al. [30] found that the progressive deterioration of left ventricular function in AMI can be alleviated by intravenous injection of MSCs, and the effects were regulated to some extent by systemic anti-inflammatory activity. In our investigation, CS-IGF-1C hydrogel greatly reduced the effect of H_2_O_2_ on hP-MSCs and NMVCs and downregulating the expression of Bad gene in vitro. In addition, CS-IGF-1C hydrogel could provide a conducive microenvironment to mediate hP-MSC activity at the molecular level, thus reducing the inflammatory responses. For instance, in vivo experiments, hP-MSCs delivered with CS-IGF-1C hydrogel reduced the expression of genes associated with inflammatory response (such as TNF-α) and reduced inflammatory cells in injured tissues. Chen et al. [31] reported that cytokines, such as tumor necrosis factor-alpha, can mediate paracrine endothelial natriuretic peptide/cGMP signaling and modulate myocardial injury and early inflammation after AMI.

It has been proved that stem cell therapy could reduce myocardial ischemia-reperfusion injury after MI and play a proangiogenic role in the recovery of damaged myocardium. Many studies have concluded that the therapeutic effects of MSCs were mainly achieved through paracrine effects and the expression of proangiogenic cytokines after transplantation. Chen et al. [32] found that endothelial progenitor cell–derived extracellular vesicles injected into ischemic myocardium through injection of hydrogel could enhance the angiogenesis and myocardial hemodynamics in the infarcted rat model. Angiogenic growth factor VEGF is thought to activate every step of proangiogenic endothelial cell behavior, such as endothelial cell invasion, migration, and attachment [33]. In our investigation, CS-IGF-1C hydrogel and hP-MSCs co-transplantation could significantly enhance HUVEC migration in vitro. In addition, in vivo experiments, hP-MSCs delivered with CS-IGF-1C hydrogel could enhance microvascular density in the ischemia cardiac and stimulate the expression of angiogenic-related genes (such as VEGF). These results indicate that CS-IGF-1C hydrogel could enhance the proangiogenic activities of hP-MSCs and promote the angiogenesis of damaged myocardium.

The use of non-invasive imaging methods to monitor the fate of transplanted cells will provide great help for the successful clinical application of regenerative therapies based on stem cell transplantation. In this study, BLI was used to monitor the retention of transplanted cells. It can be seen from the images that the hydrogel obviously prevented the loss of the transplanted cells, which may be due to the certain viscosity and mechanical properties of the hydrogel. Our BLI results have shown that the application of CS-IGF-1C hydrogel can significantly improve the survival of transplanted hP-MSCs. In addition, BLI technology also has drawbacks, such as its low resolution (about 1–2 mm) due to the low photon energy [34]. Moreover, the tissue covering the target organ will increase light attenuation and scattering, resulting in inaccurate BLI signals [35].

In this study, the reasons we used hP-MSC to transplant in C57/BL-6 background transgenic mice are as follows: On the one hand, C57/BL-6 mice are widely used as transgenic mice to mimic human immunodeficiency diseases. On the other hand, for the placenta-derived hP-MSCs, they have low immunogenicity due to their naive immune cells and low functional activity. This study has several unavoidable limitations. First, MI model is known to suffer from inconsistency and mild, intermediate, and severe injuries should be all considered. Second, during the echocardiographic examinations, there was a possibility that the MI mice had different physiological states. Further studies with a larger sample size should be warranted to confirm our findings and to evaluate the therapeutic effect of hP-MSCs incorporated with CS-IGF-1C hydrogel in mice with different types of cardiovascular disease.

## Conclusion

In summary, CS-IGF-1C hydrogel could serve as an effective niche for cell-matrix anchorage and greatly increase retention and survival of transplanted hP-MSCs in mouse MI model, which could prolong the therapeutic effect of hP-MSCs by promoting angiogenesis, anti-apoptosis, and inhibiting fibrosis. The preliminary results of our study provide further supportive evidence for the use biomaterials as vehicles for stem cell treatment of myocardial infarction.

## Supplementary information

**Additional file 1: Table S1.** RT-PCR primer sequences (human).

**Additional file 2: Table S2.** RT-PCR primer sequences (mouse).

**Additional file 3: Figure S1.** Characterization of CS-IGF-1C hydrogel and anti-apoptotic in NMVCs. (A) IGF-1C was grafted onto CS by a click reaction between the azide of IGF-1C-N3 and the alkyne of alkynyl-CS. **(B)** The thermosensitive CS-IGF-1C hydrogel neutralized with β-GP were liquid at 4 °C and cross-linked into hydrogel at 37 °C. **(C)** The protective effects of CS-IGF-1C hydrogel and hP-MSCs co-transplantation on NMVCs.

## Data Availability

All data generated and/or analyzed during this study are available from the corresponding author upon reasonable request.

## Abbreviations

AMI: Acute myocardial infarction
BLI: Bioluminescence imaging
CCK-8: Cell Counting Kit-8
CS: Chitosan
DMEM: Dulbecco’s modified Eagle’s medium
ECM: Extracellular matrix
FBS: Fetal bovine serum
Fluc: Firefly luciferase
GFP: Green fluorescent protein
HE: Hematoxylin and eosin
hP-MSCs: Human placenta–derived MSCs
HUVECs: Human umbilical vein endothelial cells
H_2_O_2_: Hydrogen peroxide
IGF-1C: Insulin-like growth factor-1C
LAD: Left anterior descending
LV: Left ventricular
LVEF: Left ventricular ejection fraction
LVFS: Left ventricular fraction shortening
LVIDd: Left ventricular internal diameter at end-diastole
LVIDs: Left ventricular internal diameter at end-systole
MSCs: Mesenchymal stem cells
NMVCs: Neonatal mouse ventricular cardiomyocytes
NEAA: Non-essential amino acid
PI: Propidium iodide
ROI: Regions of interest
SPF: Specific pathogen free
SEM: Standard error of the mean
β-GP: β-Glycerophosphate
