# Correction to: IGF-1C domain–modified hydrogel enhanced the efficacy of stem cells in the treatment of AMI

**DOI:** 10.1186/s13287-020-01685-9

**Published:** 2020-05-20

**Authors:** Yong Yao, Liang Yang, Li-feng Feng, Zhi-wei Yue, Nian-huan Zhao, Zongjin Li, Zuo-xiang He

**Affiliations:** 1 Nankai University School of Medicine, Tianjin, China; 2 Department of Nuclear Medicine, The 2nd Clinical Medical College (Shenzhen People’s Hospital) of Jinan University, Shenzhen, Guangdong China; 3 Department of Pharmacology, School of Medicine, Nankai University, Tianjin, China; 4 The Key Laboratory of Bioactive Materials, Ministry of Education, the College of Life Science, Nankai University, Tianjin, China; 5 Department of Nuclear Medicine, Huangshi Central Hospital, Affiliated Hospital of Hubei Polytechnic University, Edong Healthcare Group, Huangshi, China; 6 Hubei Key Laboratory of Kidney Disease Pathogenesis and Intervention, Huangshi, China; 7 Henan Key Laboratory of Medical Tissue Regeneration, Xinxiang Medical University, Xinxiang, China; 8 Department of Nuclear Medicine, Beijing Tsinghua Changgung Hospital, School of Clinical Medicine, Tsinghua University, Beijing, China

**Correction to: Stem Cell Res Ther 11, 136 (2020)**

**https://doi.org/10.1186/s13287-020-01637-3**

The original article [1] contained an error mistakenly carried forward by the production team that handled this article whereby Fig. 4 was duplicated over Fig. 3. The original article has now had Fig. 3 amended accordingly.
