# Supplementary material for: IGF-1C domain–modified hydrogel enhanced the efficacy of stem cells in the treatment of AMI
Source: Stem Cell Res Ther. 2020 Mar 26;11:136. doi: 10.1186/s13287-020-01637-3 (PMC7098145; doi:10.1186/s13287-020-01637-3)
Supplement: Supplementary file 1 — Additional file 1: Table S1. RT-PCR primer sequences (human). [file 13287_2020_1637_MOESM1_ESM.docx]

**Supplementary Table 1. RT-PCR primer sequences (human).**

| **Gene** | **Forward(5'to 3’)** | **Reverse(5'to 3’)** |
| --- | --- | --- |
| GAPDH | GGAGCGAGATCCCTCCAAAAT | GGCTGTTGTCATACTTCTCATGG |
| Bad | CCCAGAGTTTGAGCCGAGTG | CCCATCCCTTCGTCGTCCT |
| Bax | CCCGAGAGGTCTTTTTCCGAG | CCAGCCCATGATGGTTCTGAT |
