# Supplementary figures and images for: IGF-1C domain–modified hydrogel enhanced the efficacy of stem cells in the treatment of AMI
Source: Stem Cell Res Ther. 2020 Mar 26;11:136. doi: 10.1186/s13287-020-01637-3 (PMC7098145; doi:10.1186/s13287-020-01637-3)

**A**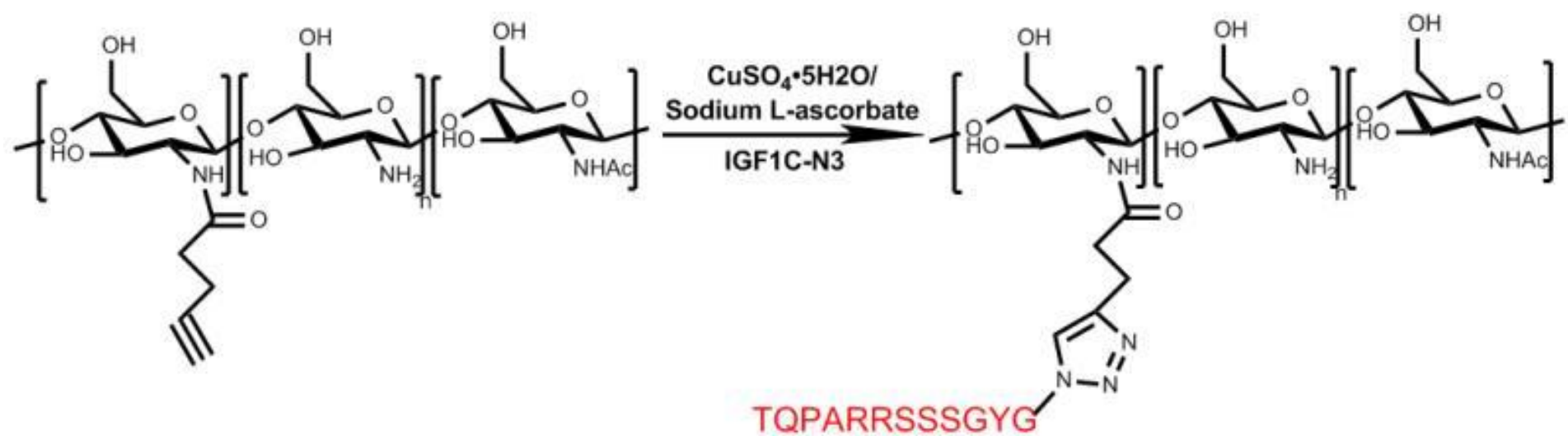**B****4°C****37°C**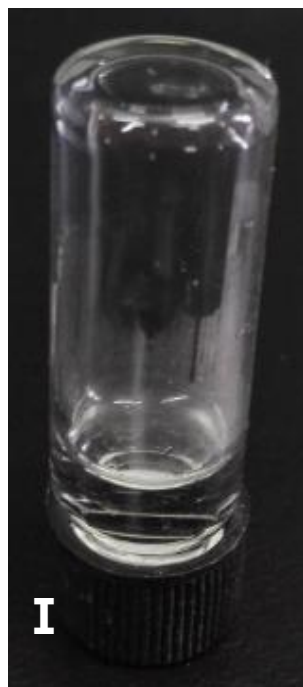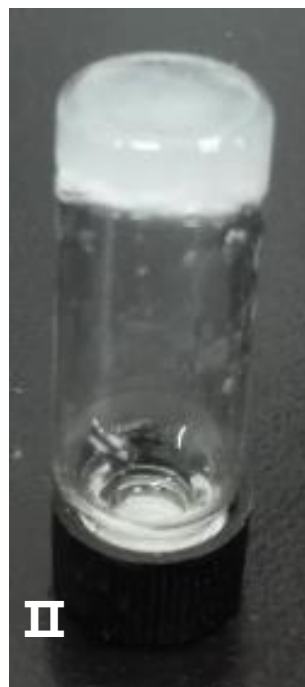**C****H<sub>2</sub>O<sub>2</sub>+PBS**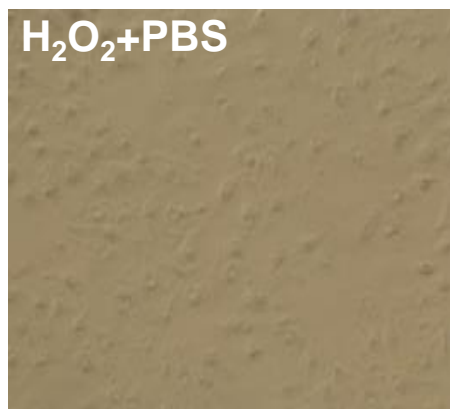**H<sub>2</sub>O<sub>2</sub>+SC**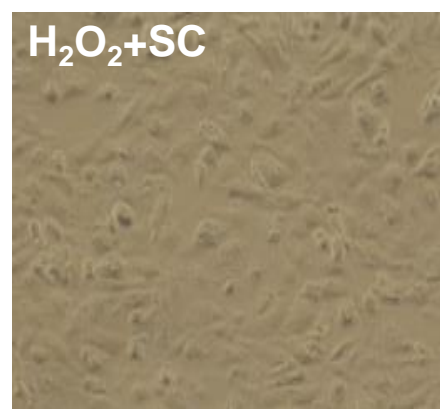**H<sub>2</sub>O<sub>2</sub>+SC+CS**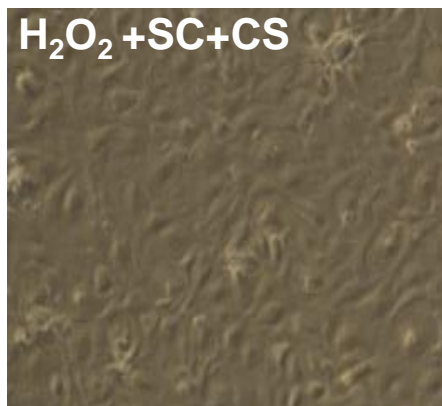**H<sub>2</sub>O<sub>2</sub>+SC+IGF**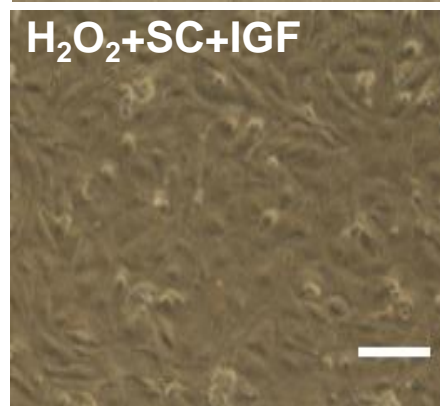

Supplement: Supplementary file 3 — Additional file 3: Figure S1. Characterization of CS-IGF-1C hydrogel and anti-apoptotic in NMVCs. (A) IGF-1C was grafted onto CS by a click reaction between the azide of IGF-1C-N3 and the alkyne of alkynyl-CS. (B) The thermosensitive CS-IGF-1C hydrogel neutralized with β-GP were liquid at 4 °C and cross-linked into hydrogel at 37 °C. (C) The protective effects of CS-IGF-1C hydrogel and hP-MSCs co-transplantation on NMVCs. [file 13287_2020_1637_MOESM3_ESM.pdf]
